# Therapeutic approaches for corneal neovascularization

**DOI:** 10.1186/s40662-017-0094-6

**Published:** 2017-12-10

**Authors:** Sepehr Feizi, Amir A. Azari, Sharareh Safapour

**Affiliations:** Ophthalmic Research Center, Labbafinejad Medical Center, Shahid Beheshti University of Medical Sciences, Tehran, 16666 Iran

**Keywords:** Corneal neovascularization, Angiogenesis, Angiogenic therapies

## Abstract

Angiogenesis refers to new blood vessels that originate from pre-existing vascular structures. Corneal neovascularization which can lead to compromised visual acuity occurs in a wide variety of corneal pathologies. A large subset of measures has been advocated to prevent and/or treat corneal neovascularization with varying degrees of success. These approaches include topical corticosteroid administration, laser treatment, cautery, and fine needle diathermy. Since the imbalance between proangiogenic agents and antiangiogenic agents primarily mediate the process of corneal neovascularization, recent therapies are intended to disrupt the different steps in the synthesis and actions of proangiogenic factors. These approaches, however, are only partially effective and may lead to several side effects. The aim of this article is to review the most relevant treatments for corneal neovascularization available so far.

## Background

A normal transparent cornea is essential to provide an appropriate anterior refractive surface, and maintaining corneal avascularity is a vital characteristic of corneal physiology. Corneal disease is the third most common cause of blindness worldwide, and corneal neovascularization is present in most affected cases [1]. The results of one study demonstrated that angiogenesis can be observed in 19.9% of human corneal buttons excised during corneal transplantation [2]. It is estimated that 1.4 million people develop corneal neovascularization per year, 12% of whom suffer the subsequent loss of vision [1].

Abnormal, new grown blood vessels in corneas sprout from pre-existing pericorneal vascular structures. Corneal neovascularization, which is a nonspecific response to different clinical insults than a diagnosis, occurs in a wide variety of corneal pathologies including congenital diseases, contact lens-related hypoxia, inflammatory disorders, chemical burns, limbal stem cell deficiency, allergy, trauma, infectious keratitis, autoimmune diseases, and corneal graft rejection [3]. These pathologies lead to a disequilibrium between proangiogenic and antiangiogenic factors that can result in the proliferation and migration of vascular endothelial cells into the corneal stroma [4, 5]. Neovascularization is a frequent complication of corneal infection, and the prevalence of infectious keratitis reflects a general picture of the extent of corneal neovascularization that occurs worldwide. Approximately, 15% of world blindness (6 million people) are caused by chlamydial infections and 146 million cases have an active infection [6]. Onchocerciasis infection is another significant cause of blindness caused by corneal neovascularization, and is reported to have blinded approximately 270,000 cases, with 120 million people worldwide at risk [6]. Herpetic keratitis is estimated to affect 500,000 cases in the USA [1]. Corneal neovascularization is also a feature of contact lens wear, particularly extended-wear usage of soft hydrogel lenses, with 1.3% of 9 million contact lens wearer estimated to have new corneal vessels [7]. Neovascularization is part of the repair of extensive chemical damage to the cornea. The prevalence of neovascularization caused by all types of chemicals (varnish removers, dyes, acids, and alkali) is approximately 37,000 cases in the USA [1].

Corneal neovascularization can lead to a decrease in visual acuity because of oedema, persistent inflammation, intrastromal protein and lipid deposition, and scarring. Moreover, a strong association between the vascularization of the recipient cornea and corneal graft rejection has been reported with the increasing risk as more areas are affected by vessels [8–12]. The formation of corneal vessels is also associated with corneal lymphangiogenesis, which enables the exit of antigens and antigen presenting cells to the regional lymph nodes [13]. There is a higher chance of lymphatic vessels being present in heavily vascularized corneas compared with mildly vascularized corneas [14]. Corneal lymphatics in vascularized recipient corneal beds adjacent to transplanted tissue can promote graft rejection through enhancing the traffic of graft-derived antigen to regional lymph nodes [15, 16].

## Review

A PubMed review was performed, analysing all publications from 1968 to 2017 concerning the topic “corneal neovascularization” (keywords: cornea, new vessels, neovascularization, and angiogenesis). Animal and human studies, published in English (full text), were included for this review.

### Pathogenesis

**Fig. 1:**
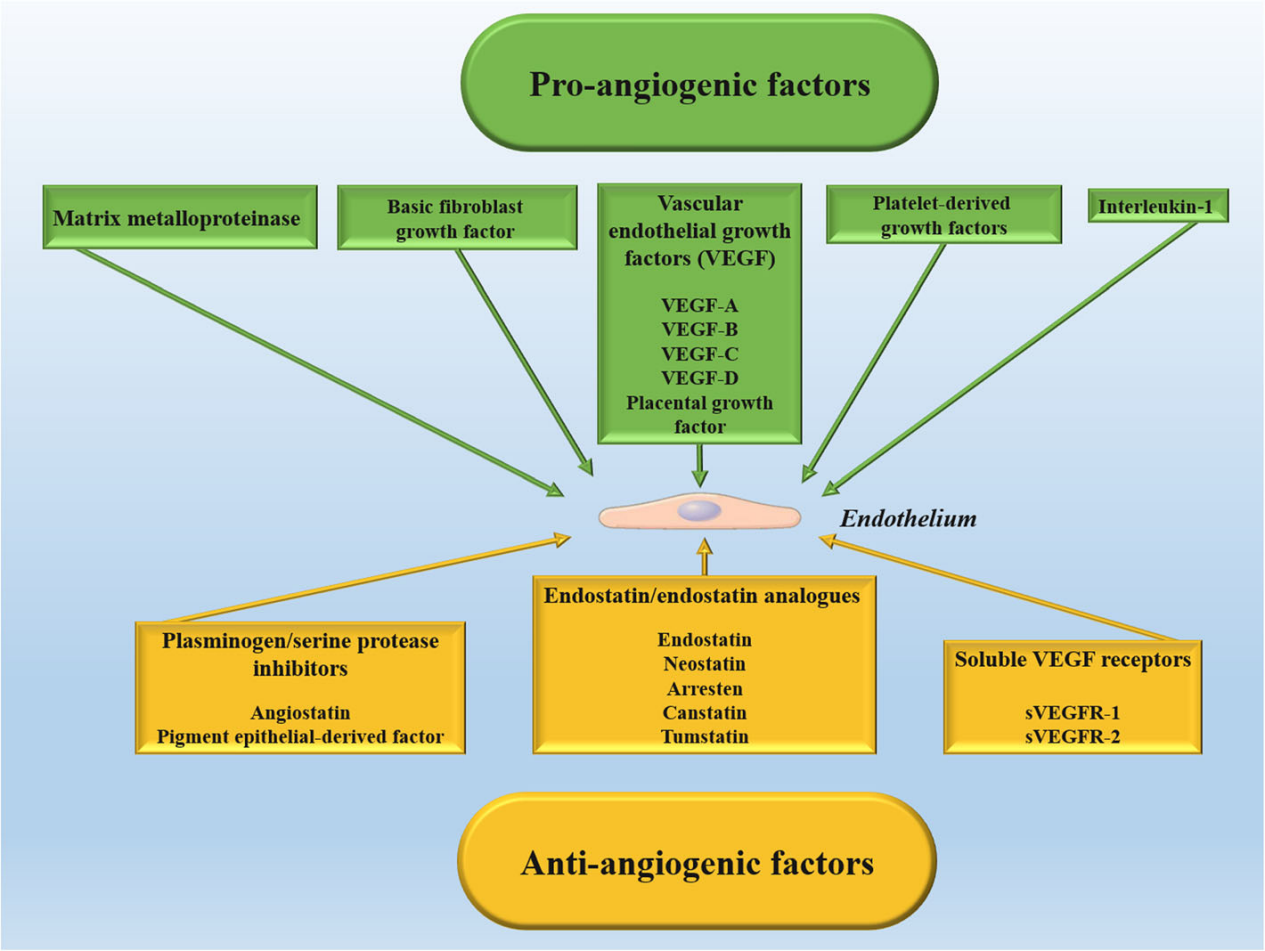
Proangiogenic and antiangiogenic factors that influence neovascularization (for details, see text)

Corneal avascularity, also called corneal angiogenic privilege, results from a balance between naturally present proangiogenic and antiangiogenic factors (Fig. 1). This angiogenic privilege is an active process. Central molecular pathways prevailing in the processes of corneal neovascularization appear to be shared among various situations and include an imbalance between proangiogenic and antiangiogenic agents, leading to an excess of proangiogenic stimuli.

Angiogenic chemical mediators consist of vascular endothelial growth factor (VEGF), matrix metalloproteinase (MMP), basic fibroblast growth factor (bFGF), platelet-derived growth factors (PDGFs), and interleukin-1 (IL-1) [17, 18]. The so-called VEGF family include VEGF-A, VEGF-B, VEGF-C, VEGF-D, and placental growth factor in mammals [19]. VEGF-A is the most significant member of the VEGF family and is secreted by a wide variety of heterogeneous cells, such as macrophages, T-cells, fibroblasts, pericytes, astrocytes, retinal pigment epithelial cells, and corneal cells (epithelium, keratocytes, and endothelium) [20]. Macrophages, stimulated by inflammation or injury, can also secrete VEGF-C and VEGF-D in the corneal stroma [21]. VEGF-A propagates its effect by interacting with tyrosine kinase receptors; VEGFR-1 and VEGFR-2. VEGFR-1 is a transmembrane receptor tyrosine kinase, whereas VEGFR-2 is a major signalling receptor for VEGF that prompts the proliferation and migration of vascular endothelial cells [22]. Lymphangiogenesis can be stimulated by VEGF-C and VEGF-D through interaction with VEGFR-3 [23, 24].

VEGF is not the only biological molecule playing the roles of hemangiogenesis and lymphangiogenesis. Other factors associated with corneal neovascularization are PDGFs that are involved in tissue remodelling, cell growth and division, and angiogenesis. It has been demonstrated that the interactions of ligands, such as PDGF-A and PDGF-B, with their corresponding receptors (PDGFR-a and PDGFR-b), are associated with corneal neovascularization [25, 26]. bFGF promotes corneal angiogenesis via its effects on VEGF-A, VEGF-C, and VEGF-D production [27]. MMP14 interacts with VEGFR1 and its enzymatic activity is essential for VEGFA-induced angiogenesis [18]. IL-1 is a proinflammatory molecule produced by different cells, including fibroblasts, macrophages, and neutrophils, and induces the expression of adhesion molecules, chemokines, and growth factors that lead to neovascularization [28].

Antiangiogenic factors can be categorized into endostatin/endostatin analogues (endostatin, neostatin, arresten, canstatin, and tumstatin), plasminogen/serine protease inhibitors (angiostatin and pigment epithelial-derived factor [PEDF]), and soluble VEGF receptors [29–32]. Angiostatin is an endogenous antiangiogenic factor that is cleaved from plasminogen. This factor is also produced in the cornea and can attach to several surface proteins in vascular endothelial cells and hinder their migration and tubule formation [33, 34]. The depletion of angiostatin after application of anti-angiostatin antibodies could enhance corneal neovascularization after excimer laser keratectomy in an animal model [35]. The implantation of an angiostatin pump could also reduce corneal neovascularization in an alkali-induced model [36].

Soluble VEGF receptors can block the effect of VEGF ligand by trapping VEGF and preventing its attachment to membrane-bound VEGF receptors. sVEGFR-1, the soluble truncated form of VEGFR-1, has a high affinity to VEGF-A and is necessary for the maintenance of corneal avascularity during development [31]. Similarly, sVEGFR-2 is the soluble form of VEGFR-2. The increased expression of sVEGFR-2 could inhibit lymphangiogenesis and enhance corneal graft survival by blocking VEGF-C in a suture-induced model [32]. These observations suggest that overexpression of soluble VEGF receptors can be exploited for inhibiting lymphangiogenesis and hemangiogenesis [37].

### Paraclinical evaluation of corneal vascularization

As new intervention modalities become available for treating corneal neovascularization, a comprehensive clinical assessment of corneal vessels, including the level of corneal vascularization, the number of quadrants involved, and the state of vessel activity is crucial for treatment planning. Additionally, the objective evaluation of abnormal corneal vessels is important for monitoring natural course and treatment response. Corneal angiography, using fluorescein and indocyanine green, provides details of the neovascular complexes, thus enabling accurate evaluation of corneal vessel anatomy and activity [38]. Fluorescein angiography determines the vessel leakage activity and maturity, whereas indocyanine green angiography provides better depiction of capillaries and deeper vessels, especially in the presence of corneal scarring [38]. Indocyanine green angiography also helps to successfully localize and differentiate the origins and extent of corneal vessels [39]. Corneal angiography, however, is time consuming, invasive, and carry the risk for uncommon, but serious adverse reactions [40, 41]. Additionally, angiography cannot determine the depth of the neovascular complexes in relation to the associated corneal pathology.

Optical coherence tomography (OCT) angiography may offer an additional non-invasive complementary approach [42, 43]. OCT systems are now able to rapidly acquire high-resolution scans over a three-dimensional (3D) volume to reconstruct coronal sections, producing an ‘en face’ view of the scanned area. Additionally, it delineates pathological corneal vessels in various conditions and allows for the appreciation of the depth of the invading vessels in relation to the associated corneal pathology [42, 43]. These features are valuable when planning for surgical interventions such as anterior lamellar keratoplasty or diathermy. This new imaging system, however, has some limitations. First, it is unable to distinguish between afferent and efferent vessels [42, 43]. Second, the current OCT angiography methods are limited in resolution to an axial and lateral resolution of approximately 5 mm and 20 mm, respectively, so that components of small vessels such as capillary loops may not be discernible [44]. In addition, OCT relies on red blood cell movement and is therefore not sensitive to acellular flow, in particular, leakage [44, 45].

## Treatments

Several approaches including amniotic membrane transplantation, topical nonsteroidal anti-inflammatory and corticosteroid medications, argon and yellow dye laser photocoagulation, photodynamic therapy, cautery, and fine needle diathermy have been advocated to shut new corneal vessels. More recently, the advent of anti-VEGF antibodies has led to a surge of interest in using these agents for the management of corneal angiogenesis. These treatments, however, have partial efficacy and may lead to a multitude of side effects (Table 1). The following sections review the current approaches for corneal neovascularization and their complications. These data are derived from studies on either animal models or human corneas (Table 2).

**Table 1:** Current approaches for the management of corneal neovascularization; advantages, limitations, and complications

Treatment	Advantages	Limitations	Complications
Corticosteroids	Reduction in inflammation and corneal neovascularization	Limited effects on pre-existing mature corneal vessels	Superinfection, glaucoma, and cataract formation
Laser	Simple and tolerable procedure, obliteration of corneal efferent vessels	Frequent reopening of the afferent vessels, ineffective in extensive corneal neovascularization	Inadvertent damage to the corneal endothelium or crystalline lens, and suture lysis
Fine needle diathermy	Inexpensive, obliteration of afferent and efferent vessels at different corneal depth	Reopening of the afferent vessels necessitating retreatment	Corneal micro perforation, intracorneal haemorrhages, transient opacification of the cornea, and striae
Anti-VEGF agents	Effective on active young vessels	Expensive, limited anti-angiogenic effects on stable mature and deep vessels, difficulties in manufacturing, short half-lives	Persistent epithelial defects and stromal thinning with topical bevacizumab

*VEGF* = vascular endothelial growth factor

**Table 2:** Animal and human studies evaluating various treatments for corneal neovascularization

Intervention	Animal studies	Human studies
Immunomodulation	Lu et al. [28], Zapata et al. [48], Cejkova et al. [49], Park et al. [50]	Cursiefen et al. [47], Ey et al. [51]
Laser treatment	Bucher et al. [56], Sidhu et al. [59]	Baer and Foster [52], Marsh [54], Kumar et al. [55], Sheppard et al. [58]
Fine needle diathermy	–	Faraj et al. [60], Romano et al. [62, 64], Spiteri et al. [63]
Anti-VEGF antibodies	Avisar et al. [75], Lee et al. [76], Lin et al. [78], Ozdemir et al. [80], Kim et al. [81], Bucher et al. [85], Liarakos et al. [86], Kim et al. [87], Akar et al. [88], Dursun et al. [89], Sener et al. [90], Oliveira et al. [92]	Dastjerdi et al. [65], Ferrari et al. [66], Krizova et al. [68], Koenig et al. [77], Chu et al. [79], Kim et al. [82]
Tyrosine kinase inhibitors	Senturk et al. [95], Saishin et al. [96], Onder et al. [97], Pérez-Santonja et al. [98], Kaya et al. [99]	–
Other treatments	Chen et al. [101], Duh et al. [102], Mori et al. [103], Jin et al. [105], Chaoran et al. [106], Dell et al. [107], Berdugo et al. [108], Cloutier et al. [110]	Cursiefen et al. [111, 112]
Combination therapy	Aydin et al. [113], Murata et al. [114], Ozdemir et al. [115], Hoffart et al. [116], Kim et al. [120]	Gerten [117], Hussain and Savant [118], Elbaz [119]

*VEGF* = vascular endothelial growth factor

### Immunomodulation

Corneal neovascularization almost always represents a state of disease that is usually associated with an inflammatory response. Therefore, inflammation has a crucial role in corneal neovascularization; accordingly, with the help of topical and periocular steroids, inflammation and consequent corneal neovascularization diminishes effectively. These medications are popular and can be used in various disease conditions. Nevertheless, the downsides of long-term use of corticosteroids are superinfection, glaucoma, and cataract formation [46]. Moreover, steroids have only limited anti-angiogenic effects and cannot effectively reduce pre-existing mature corneal neovascularization [47]. Rapamycin effectively reduced corneal angiogenesis and necrosis in a model of HSV-1 stromal keratitis [48]. Cyclosporine A-loaded nanofibers have been reported to decrease corneal vascularization caused by alkali injury in a rabbit model [49]. A study demonstrated that topical administration and subconjunctival injection of tacrolimus reduced corneal angiogenesis in a rabbit model with an effect similar to subconjunctival bevacizumab [50]. IL receptor antagonism (ra) was theorized to treat corneal neovascularization. IL-1ra is a soluble receptor inhibitor that binds to IL-1 and induces a protein sink [28]. It binds with similar affinity to IL-1α and IL-1β to neutralize intracellular signalling that is thought to act via downstream activation of VEGF and inducible nitric oxide synthase [28]. It has been shown that IL-1ra has possible therapeutic application as a topical antiangiogenic agent [28]. Other anti-inflammatory agents including nonsteroidal anti-inflammatory drugs (NSAIDs) and methotrexate are generally ineffective in controlling corneal angiogenesis as these treatments do not antagonize growth factors that induce angiogenesis [51].

### Laser treatment

Corneal vascularization can effectively be obliterated using laser therapy. This procedure is a simple outpatient procedure and is well tolerated. The argon laser [52] and 577-nm yellow dye laser [53] have been employed efficiently for the treatment of corneal vascularization in graft rejection and lipid keratopathy. Laser photocoagulation facilitates obliteration of corneal efferent vessels because these wide vessels have a relatively slower blood flow. On the contrary, it is more difficult to obliterate the afferent vessels because these vessels are thinner and deeper, and have a fast blood flow. Thus, reopening of the treated afferent vessels occurs in a high percentage of cases, necessitating multiple treatments. In the event of extensive corneal neovascularization, laser photocoagulation may be ineffective [52].

Inadvertent damage to the corneal endothelium or crystalline lens and suture lysis can occur during laser therapy of vascularized corneas. Other side effects include corneal haemorrhage and thinning, crystalline deposits on the iris, iris atrophy, and peaking of the pupil [54]. Corneal haemorrhage usually resolves without any treatment and peaking of the pupil and iris excavation are almost indiscernible after 6 to 8 weeks [55].

Photodynamic therapy (PDT) has been used to successfully obliterate corneal neovascularization safely in animals and humans. The results of an animal study showed that corneal PDT after an intrastromal injection of the photosensitizer verteporfin could selectively induce regression of lymphatic vessels without influencing blood vessels [56]. PDT generates reactive oxygen species that destroy endothelial cells and vascular basement membrane to result in vessel thrombosis and architectural remodelling. This minimally invasive treatment leads to the obliteration of the neovascular network with no damage to the surrounding healthy tissue, but several sessions may be required. Additionally, it has minimal systemic effects, making it safe when multiple sessions are needed [57, 58].

A recent study reported that frequency-doubled Nd:YAG (532 nm) laser photocoagulation is an effective treatment that can decrease the area of corneal vascularization without causing any significant side effects [55]. Kumar et al. [55] reported a reduction of 7.01% and 44.08% in the area of corneal opacity and neovascularization, respectively, following frequency-doubled Nd:YAG (532 nm) laser photocoagulation at the end of 3 months follow up. An animal study showed that the femtosecond laser can be used for the management of corneal neovascularization with minimal collateral damage [59]. The cost of the equipment and lack of availability in most centres, however, limits the widespread use of this treatment.

### Fine needle diathermy

Fine needle diathermy (FND), which can serve as an alternative to laser treatment, is an inexpensive and useful procedure for the management of corneal neovascularization. This simple procedure can be performed under topical anaesthesia and equally obliterates afferent and efferent vessels at different corneal depths. To obtain the desired result, however, retreatment may be needed [60]. A possible serious side effect is corneal micro perforation that can occur during passaging of the needle, especially in thin vascularized corneas [60]. Other potential adverse events include intracorneal haemorrhages, transient opacification of the cornea, and striae. These complications are reversible [60]. Intracorneal haemorrhage, which is the most prevalent complication, occurs intraoperatively or immediately postoperatively and resolves over a couple of weeks leaving behind crystalline deposits in some cases [60]. Transient whitening of the cornea occurs in the stroma adjacent to the needle and completely resolves after 24 to 48 h [60].

The long-term consequences of FND on limbal epithelial stem cell function and local endothelial cell survival are not yet fully understood. This technique can be associated with the extensive application of thermal energy to all corneal structures including the deep peripheral corneal stroma, potentially damaging the corneal endothelium beneath the treated area as well as corneoscleral limbus [38]. Additionally, FND can stimulate further vascularization through the secondary release of proangiogenic factors [61]. It is, therefore, logical to minimize the application of diathermy to the cornea. Recently, angiography-guided FND was used to treat afferent vessels [62, 63]. This approach can selectively treat feeder vessels with minimal thermal energy applied to the cornea and reduced risk of stromal haemorrhage [64].

### Corneal antiangiogenesis target therapies

The recent use of anti-VEGF agents has provided encouragement in the management of corneal neovascularization [65]. There are vast numbers of therapeutic agents that target the VEGF/VEGFR2 complex. These agents can generally be classified as neutralizing antibodies and kinase inhibitors. Newly formed vessels, which usually indicate an underlying ongoing pathology continuing to induce further angiogenesis, are probably best treated with anti-VEGF agents. In contrast to active young vessels, stable mature vessels in chronic neovascularization are less affected by VEGF blockade because these vessels are covered by pericytes [66, 67]. Such coverage renders the anti-VEGF treatment less successful. In addition, deep corneal vessels are less affected by anti-VEGF agents compared to superficial vessels. It is important to note that anti-VEGF therapy is only aimed at diminishing the newly developed blood vessels and it has no effects on the underlying pathology; thus, repeating the treatment may be necessary to maintain its efficacy over an extended period [68]. Additionally, high costs, difficulties of manufacturing, short half-lives, resistance, and non-responsiveness to anti-VEGF agents are major challenges in the clinic [69–71].

#### Anti-VEGF antibodies

Anti-VEGF antibodies including off-label bevacizumab (Avastin™, Genentech), ranibizumab (Lucentis™, Genentech), and aflibercept (VEGF Trap-eye (VTE), Eylea) are presently used for the management of various retinal vascular abnormalities [72]. These drugs are different in their pharmacokinetics, structure, and molecular weight. Ranibizumab and aflibercept, which received Food and Drug Administration (FDA) approval for the treatment of neovascular age-related macular degeneration, were designed specifically for intravitreal injection.

Bevacizumab is a full-length humanized murine monoclonal IgG1 antibody with a molecular weight of 149 kDa that received FDA approval for the management of different cancers. 93% and 7% of amino acid sequences of this drug are similar to the human IgG1 and murine antibody, respectively [73]. Bevacizumab recognizes all isoforms of VEGF-A. Despite being off-label, it is used commonly as an intravitreal injection for the management of various retinal diseases [74]. Topical, subconjunctival, and intraocular application of bevacizumab can to some extent decrease corneal neovascularization, subsequently improving corneal clarity [75, 76]. It can reduce the mean vessel diameter and vascularized area by 24% and 61%, respectively [77]. Its maximal effects are observed in the early administration of topical bevacizumab in the corneal neovascularization course [78]. Subconjunctival injection of bevacizumab is also effective for the management of corneal neovascularization [79]. A comparison between subconjunctival administration and topical applications of bevacizumab demonstrated that both effectively inhibit corneal angiogenesis and reduce inflammation [80].

There are desirable VEGF functions that bevacizumab may prevent, including wound healing, corneal nerve regeneration, control of vascular tone, and formation of collateral vessels. Subsequently, there is a concern that topical but not subconjunctival bevacizumab may interfere with adhesion between the basement membrane and epithelium to result in delayed wound healing and stromal thinning progression [65, 77, 81]. These adverse effects increase with the higher dose (>1.0%) and longer duration (>1 month) of treatment of topical bevacizumab [82]. Topically administered small doses of bevacizumab would not produce serious systemic adverse reactions [83]. However, uncertainty regarding systemic absorption should be considered with subconjunctival injection of bevacizumab.

Ranibizumab, a 48 kDa recombinant humanized monovalent monoclonal antibody fragment, has VEGF-binding characteristics similar to bevacizumab and binds and inhibits all VEGF-A isoforms. Since ranibizumab is the antigen-binding Fab fragment without the Fc domain from the same antibody used to create bevacizumab, its size is approximately one-third the size of bevacizumab. Therefore, ranibizumab may have a better corneal penetration compared to the parent antibody (bevacizumab). Additionally, it has been affinity matured to improve the VEGF-A binding potential [84]. In a combined in vitro and in vivo study, ranibizumab was shown to have dual antiangiogenic mechanisms through simultaneous suppression of developing blood and lymphatic vessels, highlighting its therapeutic potential in corneal neovascularization [85]. Topical ranibizumab 1% effectively treats clinically stable corneal neovascularization with a significant decrease in vessel diameter and neovascular area, but without affecting the length of the blood vessels [66]. These findings indicate that ranibizumab mainly induces the narrowing of stable corneal neovascularization more than a reduction in their length. Subconjunctival ranibizumab significantly reduces VEGF levels in the aqueous humour and iris, suggesting a possible treatment for neovascular glaucoma [86].

Comparisons of the antiangiogenic effect between bevacizumab and ranibizumab in animal studies yielded various results. Some investigators reported no significant difference between bevacizumab and ranibizumab with subconjunctival injection [87], whereas other studies demonstrated relatively fewer new vessels [88–90], shorter blood vessels [88, 89], lower corneal haziness [89], and lower degree of inflammation [90] in the patients who received bevacizumab. However, topical ranibizumab was found to be superior to bevacizumab with respect to its onset of action and degree of efficacy in a nonrandomized clinical study [84]. The use of ranibizumab may be limited due to the current drug pricing.

A new 115 KDa recombinant fusion protein, named aflibercept, has also been introduced for the management of corneal angiogenesis. It has two main portions; the VEGF-binding components from the extracellular domains of human VEGF receptors 1 and 2 are further fused to the Fc portion of human IgG1 to form the antibody [91]. The ability of aflibercept to bind VEGF-A and VEGF-B is comparable to bevacizumab and ranibizumab, but it also interacts with other members of the VEGF family including PDGF and placental growth factor and prevents the ligand-induced activation of the receptors [91]. Oliveira et al. [92] showed that aflibercept suppresses bFGF-induced corneal neovascularization in a murine model. It has been found that aflibercept has a greater binding affinity for VEGF between 10 and 12 weeks after injection compared with bevacizumab and ranibizumab [93]. It suggests that aflibercept can be used for the treatment of corneal neovascularization in eyes previously treated with either bevacizumab or ranibizumab. In the latest in vitro study to evaluate comparative cytotoxic effect of ranibizumab, aflibercept, and bevacizumab, ranibizumab and aflibercept were found to cause less damage to the corneal epithelium in cases of pre-existing epithelial defects [94].

#### Tyrosine kinase inhibitors

Since the angiogenic activity of VEGF is mediated through its receptors, the blocking of receptor-ligand interactions is a promising approach for anti-VEGF therapies [95]. These agents impede the activity of VEGF by inhibiting tyrosine kinase in the intracellular part of the VEGF receptor [96]. Multikinase inhibitors are small molecules that inhibit VEGFR-1, VEGFR-2, and related receptor tyrosine kinases including PDGFR. In clinical experiments, both topical and systemic administration of tyrosine kinase inhibitors has been implicated. Drugs in this group include regorafenib, sunitinib, trastuzumab, lapatinib, and midostaurin. Regorafenib, which is a multikinase inhibitor, has inhibitory effects similar to topical dexamethasone and bevacizumab [97]. Sunitinib, a multitargeted receptor tyrosine kinase inhibitor, blocks both VEGF and PDGF and can decrease corneal angiogenesis more effectively than bevacizumab [98]. Compared to systemically administered trastuzumab, systemic administration of lapatinib is more effective in preventing corneal angiogenesis [99]. Midostaurin is an orally administered small multikinase inhibitor that binds to the intracellular enzymatically active portion of VEGFR and PDGFR and blocks phosphorylation and activation of the VEGF cascade. This drug was shown to inhibit choroidal neovascularization. TG100801 is another multikinase inhibitor of predominantly VEGFR-2 and other protein kinases that regulate angiogenesis. It is a topically administered prodrug that is readily converted to the active compound in the eye. Thus far, it exhibits excellent ocular pharmacokinetics and poor systemic absorption. Currently, it is being evaluated for the management of age-related macular degeneration and has potential application for corneal neovascularization [100].

### Other potential targets of therapy

The limited success of the anti-VEGF antibody therapy is due partially to the maturation of established vessels that is characterized by pericyte covering the vasculature. Therefore, it is important to target other key players in the process of angiogenesis. A recent animal study showed that galectin-3 is a key molecule that modulates the process of pathological angiogenesis, and inhibiting galectin-3 can ameliorate pathological corneal angiogenesis as well as fibrosis [101].

PEDF, a glycoprotein with anti-tumorigenic, anti-angiogenic, and neurotrophic functions, can prevent VEGF, bFGF, and interleukin-8 (IL-8/CXCL8)-mediated angiogenesis through inhibiting endothelial cell migration and inducing cell apoptosis simultaneously [102, 103]. It also contributes to the anti-angiogenic effect of the amniotic membrane [104]. A chemically-induced corneal neovascularization model demonstrated that topical PEDF or PEDF-derived (P5-2 and P5-3) peptides could downregulate VEGF expression and inhibit corneal angiogenesis [105].

PDGFs are proteins involved in cell growth, division, tissue remodelling, and angiogenesis. Ligands including PDGF-A and PDGF-B and receptors including PDGFR-a and PDGFR-b are associated with angiogenesis and can be found in corneal tissue [25, 26]. It was reported that intraperitoneal injection of AG 1296, a PDGF receptor inhibitor, led to the loss of capillary pericytes and reduction of vessel density in advanced corneal neovascularization [106, 107].

Aganirsen (GS-101) is an antisense oligonucleotide that inhibits insulin receptor substrate-1 (IRS-1) mRNA expression. Engagement of IRS-1 proteins has been demonstrated to be an important step in the process of neovascularization [108], and overexpression of IRS-1 have been reported in corneal angiogenesis [109]. Aganirsen dose-dependently inhibits IRS-1 expression and angiogenesis while reducing VEGF-A and the proinflammatory cytokine IL-1b mRNA levels [108–110]. Corneal topical application of aganirsen is well tolerated and has no side effects [111]. Cursiefen et al. reported on a series of 69 patients with keratitis-related progressive corneal angiogenesis who randomly received aganirsen (34 patients) or placebo (35 patients) [112]. Although the two groups were comparable in visual acuity, aganirsen significantly decreased the relative corneal angiogenesis area after 90 days by 26.20% which persisted after 180 days [112]. Additionally, aganirsen tended to reduce the transplantation need at day 180 [112].

### Combination therapy

There is still no clear consensus about an ideal therapeutic approach for actively growing and established corneal neovascularization. Although mainstay therapy currently comprises steroids and VEGF inhibitors, some combination therapies have been investigated with encouraging outcomes. The theory is that by combining various methods of control, the different mechanisms that sustain corneal neovascularization will be targeted and that this therapy will be more efficacious in preventing further progression. Also, the treatment may allow us to use a lower dose of each drug. Topically administered combinations of triamcinolone acetonide 10 μg/ml with doxycycline 10 mg/ml or with low molecular weight heparin 10 mg/ml have synergistic effects that can efficiently suppress corneal neovascularization [113, 114]. Low molecular weight heparins seem to diminish the binding of angiogenic agents to the corresponding receptors. Using topically administered doxycycline has also been shown to have added antiangiogenic effects [113, 114].

A combination therapy, consisting of both anti-inflammatory drugs and antiangiogenic factors may be better than monotherapy approaches. For example, the therapeutic outcome of both dexamethasone and bevacizumab or etanercept and bevacizumab as a combination therapy may be more effective than monotherapy approaches in the management of corneal neovascularization [115, 116]. Marked decrease in corneal angiogenesis can be achieved with bevacizumab in conjunction with argon laser therapy [117], FND [118, 119], and PDT [120]. In these combined treatments, argon laser photocoagulation, FND, and PDT provide a potential treatment by closing abnormal corneal vessels, whereas bevacizumab acts to prevent new angiogenesis.

## Conclusions

Corneal angiogenesis is a common endpoint in different ocular surface conditions including chemical injury, trauma, chronic contact lens wear, autoimmune diseases, infectious keratitis, and corneal graft rejection. Although corneal angiogenesis is useful in halting stromal melts, facilitating wound healing, and dissipating infections, it sacrifices corneal clarity by inducing persistent inflammation, oedema, lipid deposition, and tissue scarring, resulting in bad vision. In addition, breaching corneal immune privilege by new vessels promotes corneal graft rejection. VEGF plays an important role in the pathologic neovascularization in a huge number of eye diseases hence, it is the most important target for anti-angiogenic treatments. According to literature, anti-VEGF agents are quite effective in occluding actively growing blood vessels but not established large vessels in which surgical approaches, such as fine needle diathermy or laser photocoagulation, are invaluable.
